# Genetic Diversity and Recombination Analysis of Canine Parvoviruses Prevalent in Central and Eastern China, from 2020 to 2023

**DOI:** 10.3390/microorganisms12112173

**Published:** 2024-10-29

**Authors:** Shunshun Pan, Yuanzhuo Man, Xin Xu, Jun Ji, Shiyuan Zhang, Honghui Huang, Ying Li, Yingzuo Bi, Lunguang Yao

**Affiliations:** 1 Henan Provincial Engineering and Technology Center of Health Products for Livestock and Poultry, Henan Key Laboratory of Insect Biology in Funiu Mountain, Nanyang Normal University, Nanyang 473061, Chinalunguangyao@163.com (L.Y.); 2 College of Animal Science, South China Agricultural University, Guangzhou 510642, China

**Keywords:** canine parvovirus, recombination, evolution tree, sequence analysis, mutations

## Abstract

Canine parvovirus type-2 (CPV-2), the primary causative agent of serious canine enteric diseases, is highly contagious and associated with high fatality rates worldwide. To comprehend the current emergence of CPV-2 in central and eastern China, 130 rectal swabs from domestic or stray dogs with gastroenteritis symptoms were collected during 2020–2023. A total of 118 positive samples were detected via polymerase chain reaction, and further used to amplify and sequence the VP2 gene. Sequence analysis of the deduced amino acids of VP2 protein indicated that CPV-2c was the most prevalent variant (n = 106, 89.83%), followed by the novel CPV-2a (n = 10, 8.47%) and CPV-2b (n = 2, 1.69%) variants. The VP2 protein from the obtained and reference strains showed 86.95% (AH2103 and HB2108) to 99.94% identity. Based on the nine predicted recombination events, some prevalent CPV-2c strains were highly similar to previously isolated strains, indicating their complex evolution and recombination. The predicted analysis suggested that mutations in the antigen epitope (Val219Ile, Phe267Tyr, and Asn426Glu) and other mutations (Met87Leu, Ile101Thr, and Ser297Ala) affect the tertiary structure of the VP2 protein. This research will help us understand the recent evolution and mutation of Chinese CPV-2 and provide suggestions for updating the CPV-2 vaccine.

## 1. Introduction

Canine parvovirus type-2 (CPV-2), which was first detected in 1978 in domestic dogs, is usually recognized as a new feline panleukopenia virus with host range variation [1]. Since its discovery, CPV-2 has been detected worldwide, resulting in high infection rates in dogs [2]. CPV-2 commonly causes diarrhea, emesis, anorexia, depression, pyrexia, and hypothermia, particularly in puppies; the virus mainly resides in the intestinal crypts and lymphatic organs of diseased dogs but can be found in any organ of susceptible animals [3,4,5,6,7].

CPV-2, a member of the *Parvoviridae* family, has no membrane, 5323 bp of genomic DNA, and two open reading frames (ORFs). The first ORF encodes the nonstructural proteins NS1 and NS2, whereas the second ORF encodes the structural proteins VP1 and VP2 [8]. Of these viral proteins, VP2 protein of CPV-2 constitutes approximately 90% of the virion and is a crucial protein for determining CPV-2 antigen features, genotypes, and receptor binding [9]. CPV-2 has evolved as a variant of a feline parvovirus by acquiring mutations that allow binding to the transferrin receptor type 1, which is conserved between the canine and feline species and causes illness [1]. A previous study characterized the genotypes of CPV-2 strains based on critical amino acid (aa) residues of VP2 protein: the classical CPV-2 was defined as 87Met, 101Ile, 300Ala, 305Asp, and 375Asn [10]. Immediately after the emergence of CPV-2, a novel antigenic CPV variant (CPV-2a) emerged in 1979, with four specific aa mutations (Leu87Met, Ile101Thr, Ala300G, and Asp305Tyr), and replaced classical CPV-2 worldwide [11]. The four mutations specific for CPV-2a affected the antigenic reactivity of monoclonal antibodies and the binding affinity to the viral receptor [12]. Subsequently, CPV-2b evolved with nonsynonymous mutations involving aa 426 (Asn426Asp) and 555 (Ile555Val), which was first detected in the United States in 1984 [8]. Ser297Ala in VP2 protein distinguishes CPV-2a/2b from the new CPV-2a/2b variants, and the host’s immunological pressure on the virus leads to the formation of a new genotype of CPV-2c (Asp426Glu) [11]. These variants co-circulate with diverse manners in different geographic regions worldwide. In Asia, CPV-2a was prevalent as the dominant genotype for a long time, until replaced by CPV-2c in 2020 [11]. According to the literature, CPV-2c may substitute CPV-2a in Asia, South America, and Africa but not in Europe or Oceania [13]. Notably, a study conducted in Henan Province of China during 2020–2021 first reported a unique mutation (Ile447Met) in VP2 protein of five CPV-2c strains [2]. According to the review of CPV-2, the major recent trend in Asia, South America, North America, and Africa is the replacement of CPV-2a with CPV-2c. Ala5Gly and Gln370Arg mutations in CPV-2c-VP2 strains, which are prevalent in most regions of Asia, have become the predominant mutations, and further monitoring of the variation of the trend of CPV-2 in Asia is crucial [2,14]. In China, CPV-2a and CPV-2b strains were first detected in 2009 [15]. Since then, CPV-2a and CPV-2b have been reported to circulate in northeast China, and novel CPV-2a/2b and CPV-2c genotypes were mainly circulated in central China [7,16]. Meanwhile, some novel VP2 mutation sites (Phe267Tyr, Ser297Ala, and Tyr324Ile) with high prevalence were present in new CPV-2a, new CPV-2b, and CPV-2c; and Gln370Arg was mainly found in CPV-2c from central China [16]. These mutation sites adjacent to the genotype-determined sites in VP2 may be related to the antigenicity and pathogenicity of CPV-2 [16].

Considering the importance of VP2 in pathogenicity and immune evasion, this study focused on the molecular characterization of CPV-2 VP2 to understand the emergence of CPV-2 in central and eastern China.

## 2. Materials and Methods

### 2.1. Ethics Approval and Consent to Participate

The dog owners and the South China Agricultural University Committee for Animal Experiments approved the protocols (approved ID: SYXK 2019-0136; 8 June 2020). The experiments were conducted following the recommendations of the Guide for the Care and Use of Laboratory Animals of the National Institutes of Health.

### 2.2. Sample Collection and DNA Extraction

In this study, 130 rectal swab samples of dogs with clinical gastroenteritis were humanely obtained from pet dogs in hospitals or stray dogs in Anhui (36 samples), Henan (35 samples), Jiangsu (27 samples), and Hebei (24 samples) provinces during 2020–2023. The collected samples stored in 5 mL cryovials were homogenized in 2 mL of phosphate buffered saline (PBS) solution. Finally, 200 μL homogenate was used for DNA extraction with a viral DNA/RNA extraction kit (TransGen Biotech, Beijing, China, CAT: ER201). The extracted DNA was maintained at −20 °C until further use.

### 2.3. Detection and VP2 Sequencing

The CPV-2 specific primer pair of VP2-F (5′-AGAGACAATCTTGCACCAAT-3′) and VP2-R (5′-ATGTTAATATAATTTTCTAGGTGCT-3′) (located at 2779–4533 of canine/China/09/2016 strain; accession number: MF805797) was constructed using Primer Premier 5.0 software (Premier Biosoft, San Francisco, CA, USA). The specific primer pair synthesized by Biological Biotech (Shanghai, China) Co. Ltd. was used to amplify the entire VP2 gene (1755 bp) using polymerase chain reaction (PCR), which revealed the presence of CPV-2 DNA [17]. PCR was conducted using the PrimeScript™ High Fidelity RT-PCR Kit (Takara Biotech, Dalian, China, CAT: RR006A) under the following cycling conditions: 3 min of predenaturation at 95 °C; followed by 35 cycles of denaturation at 95 °C for 30 s, annealing at 55 °C for 30 s, extension at 72 °C for 2 min; and final extension at 72 °C for 5 min. After being cloned into the pMD™18-T Vector Cloning Kit (Takara Biotech, Dalian, China, CAT: 6011), each amplicon was sequenced via Sanger dideoxy sequencing (Syn-Biotechnology, Suzhou, China). Supplementary Table S1 shows information on all CPV-2 strains sequenced in this study. The sequencing results 100% identical to the CPV-2 vaccine strain (Pfizer vaccine strain 06, accession number: FJ197846) were screened out and not used for downstream analysis.

### 2.4. Nucleotide Identity and Amino Acid Mutations

The VP2 sequences of the obtained CPV-2 strains were assembled and aligned with the reported VP2 sequence of CPV-2 (Supplementary Table S2) using BioAider Version 1.527 [18]. For a more intuitive display, the results of identity analysis were presented on a heat map using the Chiplot website (https://www.chiplot.online/ (accessed on 25 August 2024)). Mutations in the VP2 protein between the obtained and reference CPV-2 strains from China and abroad were analyzed using MegAlign program of Lasergene v7.1 (DNASTAR, Inc., Madison, WI, USA).

### 2.5. Prediction of the Epitope and Tertiary Structure

The linear antigen epitopes of the VP2 aa sequences of all the obtained and reference strains were predicted using antigen-prediction procedure in DNAMAN (Version 9.0, USA. The mutant and conservative VP2 residues of CPV-2 were structurally analyzed of homology using SWISS-MODEL (https://swissmodel.expasy.org/interactive (accessed on 28 october 2024)), and finally post-modeling was completed via PyMOL software version 2.4.0.

### 2.6. Phylogenetic and Recombination Analyses

Using MEGA-X Version 10.1 maximum likelihood technique with model of Tamura 3 parameter add G (preferred according to the results by the model-finding procedure), a phylogenetic tree of the entire VP2 nucleotide sequences was constructed (bootstrap replicates = 1000) [19]. To assess the occurrence of recombination events, VP2 sequences of 118 obtained and 37 reference CPV-2 strains were analyzed using the RDP 4 package, and the recombinants were detected via seven algorithms, including RDP, GENECONV, Bootscan, MaxChi, Chimera, SiScan, and 3 seq in the embedded RDP 4.97 program [20]. Parental and recombinant strains involved in each recombination event were analyzed based on similarity and Bootscan using SimPlot version 3.5.1 [21]. Potential recombination events detected via three or more programs along with the similarity analysis of the parents were considered potential events, based on the highest acceptable *p*-value cutoff of 0.05 [22].

## 3. Results

### 3.1. Detection of CPV-2 and Nucleotide Identity of VP2

Of the 130 samples, 118 (90.77%) were validated as CPV-2 positive via PCR. Among these samples, the positivity rate of CPV-2 was 88.88% (32/36) in Anhui Province, 85.71% (24/28) in Hebei Province, 97.22% (35/36) in Henan Province, and 90% (27/30) in Jiangsu Province. Based on the clinical information of dogs with sequenced strains, most dogs had vaccination records in pet hospitals and still tested positive for CPV-2.

**Figure 1:**
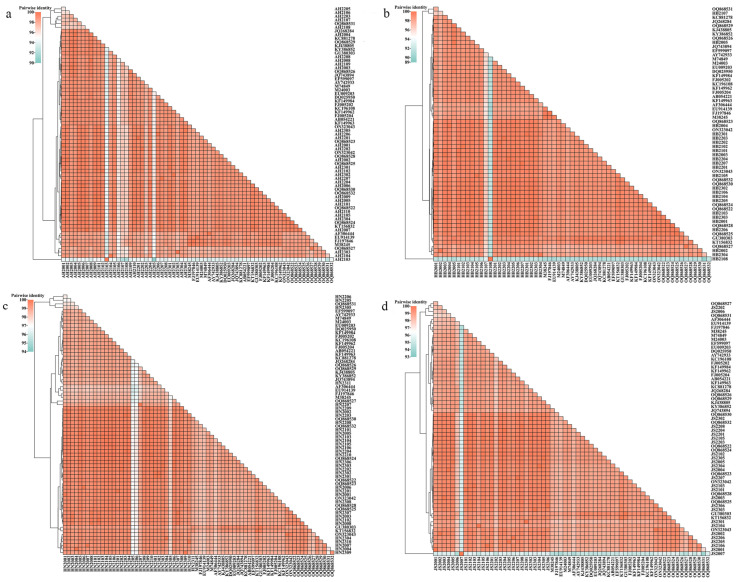
Pairwise identity plot of VP2 nucleotide sequence of the obtained and reference CPV-2 strains. (**a**) Plot: 36 strains from Anhui Province and reference strains. (**b**) Plot: 24 strains from Hebei Province and reference strains. (**c**) Plot: 35 strains from Henan Province and reference strains. (**d**) Plot: 27 strains from Jiangsu Province and reference strains. The VP2 sequences of the sequenced and reference strains shared identity of 86.95–99.94%.

The entire VP2 gene of CPV-2 strains was successfully sequenced to obtain 118 complete VP2 sequences, which indicated all the strains were not vaccine-like strains. The VP2 sequences of the sequenced and reference strains showed 86.95–99.94% identity. Compared with the reference strains, nucleotide identities of 90.66–99.94%, 89.97–99.94%, 94.99–99.94%, and 93.85–99.94% were shared by 36 CPV-2 strains from Anhui Province, 24 from Hebei Province, 35 from Henan Province, and 27 from Jiangsu Province, as displayed using a heat map in Figure 1. Notably, AH2103 (collected from Anhui, 2021) and HB2108 (collected from Hebei, 2021) shared the lowest identity of 86.95%. The identity matrix associated to Figure 1 is displayed in Supplementary Material S2.

### 3.2. Potential Epitope and Representative Amino Acid Mutation Analysis

In this study, 22 putative linear epitopes were identified through B-cell epitope prediction using the VP2 aa sequences of all strains (Table 1). The epitope with the maximum score was located at 247–256 position of the aa site.

VP2 aa sites indicated that the 10 obtained CPV-2 strains exhibited new CPV-2a genotypes, two strains exhibited new CPV-2b genotypes, and all remaining strains contained representative sites of CPV-2c genotype, suggesting that CPV-2c is the predominant variant genotype in central and eastern China during 2020–2023. Supplementary Table S3 shows the major aa mutations in the VP2 protein of 118 sequenced strains. Met87Leu, Ile101Thr, Ser297Ala, and Asn375Asp mutations were found in all obtained Chinese strains. Further, the epitopes located at 219–227, 267–276, and 421–434 contained the variant mutations in the obtained CPV-2 strains (Table 1).

### 3.3. Tertiary Structure Analysis of the VP2 Protein

**Figure 2:**
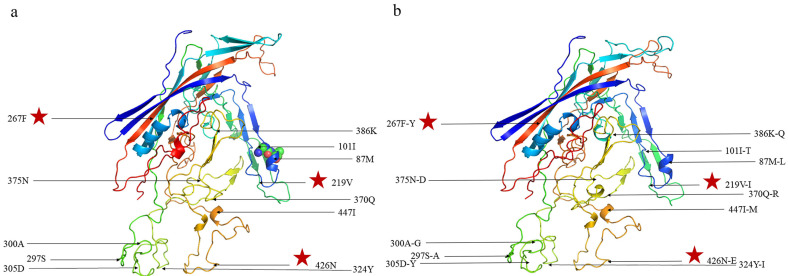
(**a**) Tertiary structure of VP2 in the CPV-2 Pfizer vaccine strain and (**b**) structural model of the CPV-2 mutant VP2 protein. Mutations located in the predicted antigen epitope are marked with a red star.

Relying on the suggested template (PDB code: 6oas.3, validated by electron microscopy [23]), the tertiary structure model and primary aa mutations predicting the conservative and variant VP2 residues are shown in Figure 2. The results revealed that Met87Leu, Ile101Thr, Ser297Ala, and Asn426Glu may affect the change in the tertiary structure of the VP2 protein.

### 3.4. Phylogenetic and Recombination Analyses

**Figure 3:**
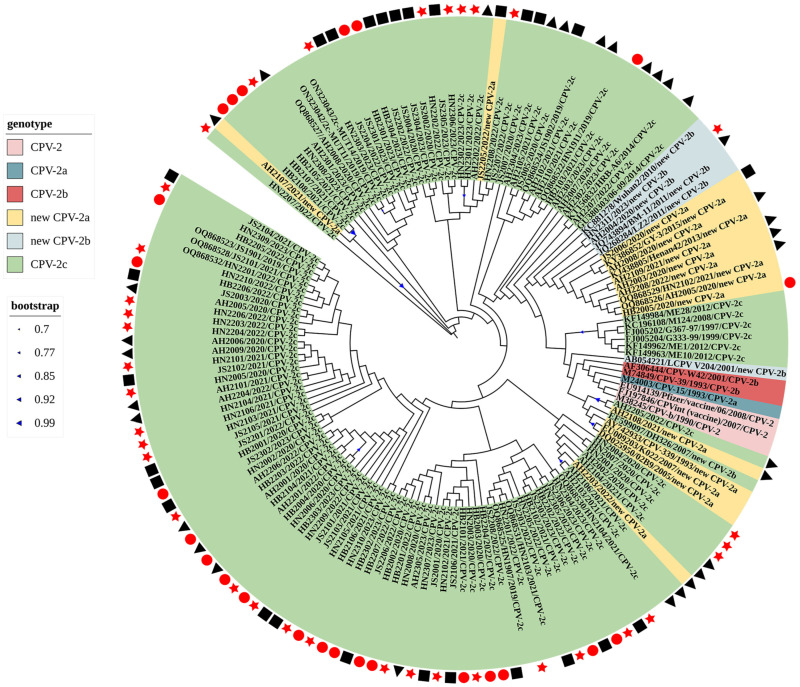
Evolutionary tree of the VP2 sequence of the obtained and reference strains. Strains from Henan Province are marked with red stars, strains from Hebei Province are marked with red circles, strains from Anhui Province are marked with black triangles, and strains from Jiangsu Province are marked with black rectangles. The phylogenetic clustering demonstrated that most of the obtained and reference CPV-2c strains were genetically close to each other.

Figure 3 presents the phylogenetic tree based on the VP2 nucleotides of the obtained and reference strains, demonstrating that most of the obtained and reference CPV-2c strains were genetically close to each other. Four new CPV-2a strains (AH2107, AH2108, AH2203, and JS2205) were not clustered with other novel reference CPV-2a strains from China and other countries (identity ranged from 95.56% to 99.94%); they were closely related to most CPV-2c strains (identity ranged from 86.95% to 99.94%). These results were consistent with the identity results, indicating that the CPV-2c strains differed between each other. Six new CPV-2a strains (AH2003, AH2008, JS2006, HB2005, AH2109, and AH2208) were closely related to some new Chinese CPV-2a strains (KY386852, KJ438805, OQ868526, and OQ868529) (identity ranged from 98.8% to 99.94%) and were distant from some new CPV-2a strains from other countries (AY742933, DQ025950, and EU009203) (identity ranged from 98.52% to 99.94%). Two new CPV-2b strains (AH2004 and HN2311) were also closely related to some newly reported Chinese CPV-2b strains (JQ268284, JQ743894, and KC881278) (identity ranged from 98.52% to 99.94%) but were distant from some new Chinese CPV-2b strains (LCPV strain, accession number: AB054221) (identity ranged from 99.37% to 99.83%) and South Korean strains (DH326 strain, accession number: EF599097) (identity ranged from 99.09% to 99.66%).

**Figure 4:**
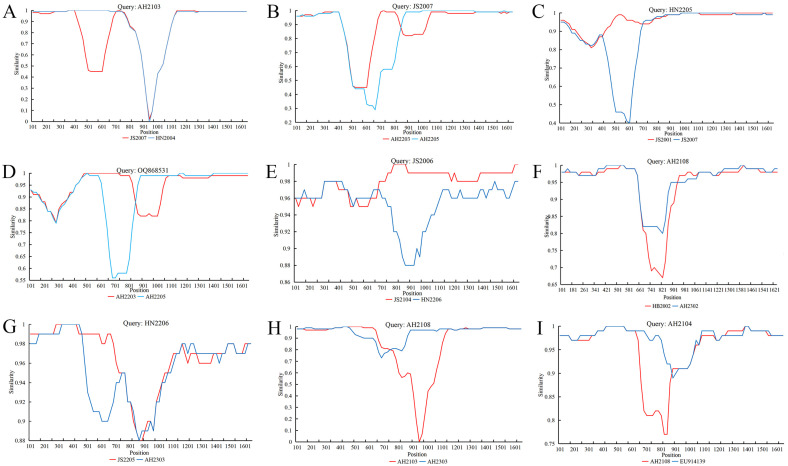
The results were verified using the SimPlot analysis of nine recombination events based on RDP4 prediction analysis. The abscissa represents the sequence positions, whereas the ordinate represents the similarity between the two recombination parents of the nine predicted recombination events. (**A**) Recombination occurrence in AH2103. (**B**) Recombination occurrence in JS2007. (**C**) Recombination occurrence in HN2205. (**D**) Recombination occurrence in OQ868531. (**E**) Recombination occurrence in JS2006. (**F**) Recombination occurrence in AH2108. (**G**) Recombination occurrence in HN2206. (**H**) Recombination occurrence in AH2108. (**I**) Recombination occurrence in AH2104.

Based on data analysis of RDP4, the average *p*-values of all detection methods of reorganization events are listed in Table 2 (each reorganization event is confirmed using no less than three detection methods), and nine recombination events that occurred in CPV-2 strains are shown in Table 3. For these recombination events, 8 out of 9 obtained CPV-2 strains were recombinated from Chinese strains from different provinces and years obtained in this study. AH2104 was predicted to be a recombinant of EU914139 (vaccine strain from the USA) and AH2108, found in event I (from nt 898 to 1999). Furthermore, the predicted results of RDP4 were simultaneously verified via further analysis using SimPlot software version 3.5.1. According to the breakpoints of recombination events indicated by the intersections in Figure 4, the breakpoints were mainly situated in the middle region of VP2 coding.

## 4. Discussion

In this study, VP2 sequences of the obtained and reference strains showed 86.95–99.94% identity, and AH2103 (collected from Anhui, 2021) and HB2108 (collected from Hebei, 2021) shared the lowest identity of 86.95%, indicating the VP2 divergence between CPV-2 strains in this study. Since the first report of the CPV-2 infection, various genotyping studies have been conducted on the epidemic features of CPV-2 in different regions [1]. The main prevalent strains in European countries are CPV-2b and CPV-2c. In Asia, the previous main prevalent genotype was CPV-2a, but this has recently been substituted by CPV-2c [2,24]. A similar situation occurred in South America, North America, and Africa. Furthermore, CPV-2c harbored the Ala5Gly and Gln370Arg mutations in VP2, which have been circulating in most of Asia and in Europe and Africa [2]. This study further revealed that CPV-2c is the most prevalent genotype in central and eastern China. Two CPV-2c strains, AH2103 (obtained from Anhui, China, 2021) and HB2108 (obtained from Hebei, 2021), shared only 86.95% identity, demonstrating that CPV-2c strains with large sequence differences co-circulated in central and eastern China. Although vaccines against CPV-2 have been used worldwide, CPV-2 infection remains common, mainly in puppies, strengthening the importance of epidemiological testing of CPV-2 variants [10]. Low immune protection is one of the key causes of continuous CPV-2 circulation worldwide, probably due to reasons such as the presence of maternal interfering antibodies, immunization failure, and circulation of multiple antigenic variants of CPV-2 [25]. Phe267Leu mutation is observed in the major antigen epitope with a high mutation rate, which may affect viral antigenicity and lead to immune escape. Therefore, it is vital to continuously investigate CPV-2 and circulation of various antigenic variants to provide technical support for China’s vaccine update.

The VP2 of strains from stray and pet dogs shared high identities, suggesting the extensive spread of CPV-2 via horizontal transmission through virus-contaminated excreta in shared spaces, parks, and public spaces. In China, human activities related to dog breeding further increase the risk of cross-regional transmission of canine viruses [26]. This may be a similar reason for the highly shared identity of CPV-2 strains between different provinces in this study.

To further explore the genetic relationship between the obtained and reference CPV-2 strains, phylogenetic and recombination analyses were performed. The VP2 phylogenetic tree revealed that the CPV-2c strains examined in this study were closely related to most previously reported CPV-2c strains. Four new CVP-2a strains were not clustered with other CPV-2a strains, indicating that the change in sporadic bases does not affect overall CPV-2 evolution [27,28]. All new CPV-2a strains sequenced in this study exhibited an affinity toward some previously reported Chinese strains (KY386852, KJ438805, OQ868526, and OQ868629) on the evolutionary tree but showed a distant relationship with reference strains (AY742933, DQ025950, and EU009203) mainly from New Zealand, France, and South Korea.

In the nine recombination events, the recombinant CPV-2 strains in various provinces across different years revealed some prevalent CPV-2 strains, suggesting the co-circulation of viruses with different genotypes and evolutionary ancestors. The complex and multiple recombination events also indicated that recombination is an important mechanism of CPV-2 evolution [11]. In recombination event-I, the Pfizer vaccine strain (accession number: EU914139) served as a potential major parent of the AH2104 strain (collected from Anhui, 2021). Additionally, AH2108 (collected from Anhui, 2021) belongs to the new CPV-2a strain and not to the same clade as the other new CPV-2a strains on the evolutionary tree. It is twice as recombinant in the predicted recombination events (F and H), revealing the complex evolution of CPV-2. Combining the results of our previous study and reports in northeast China, the main variant in eastern and central China is CPV-2c, and the main CPV-2 epidemic strain in northeast China is new CPV-2a, which may be locally determined [8,27]. Therefore, a higher number of CPV-2 strain samples are required for further verification.

To better elucidate the immune escape and structural change for CPV-2 variant strains, the epitope and tertiary structure were predicted. The sites 267 and 426 with high mutation rates in CPV-2 have antigenic significance and affect the change in the tertiary structure of VP2 [16]. Additionally, Ser297Ala in all obtained strains has been reported to induce changes in the antigenicity of CPV-2 variants and may have a significant effect on ongoing host adaptation [29]. The mutation site 426 at the antigenic epitope produces viral variants (termed as CPV-2b and CPV-2c) [12]. In addition to the previously reported Phe267Tyr mutation (115/118, 97.45%), Phe267Leu mutation in AH2011 (collected in 2020, Anhui) may affect the antigenicity change and differentiate into new subtypes [30]. Another Val219Ile mutation located at the B-cell antigen site may affect viral antigenicity [31,32]. As previously reported, aa residue 93 had an impact on CPV’s ability to bind to canine transferrin receptor, and its mutation may alter the antigenicity and host range of the virion [23]. Met87Leu, which was adjacent to aa residue 93, and Ile101Thr help CPV-2a differ from the original CPV-2. Further, Asn426Glu was a determinant site for genotyping of genotype CPV-2c. In this study, Met87Leu, Ile101Thr, Ser297Ala, and Asn426Glu were all predicted to affect the change in the tertiary structure of VP2 protein. These mutations may cause differences in the antigenicity and even in the recognition of cellular receptors. Further experiments are warranted to determine the specific biological function related to the emerging mutation sites.

## 5. Conclusions

In summary, this study revealed that CPV-2c was the most prevalent genotype of CPV-2 in eastern and central China. Some mutations in the predicted antigen epitope may affect the tertiary structure of the VP2 protein. Multiple recombinants potentially occurred in the obtained CPV-2 strains parented from strains from different Chinese provinces and years. These results suggest that continued identification of the mutation and recombination in VP2 protein may contribute to understanding the evolution of parvovirus diversity.

## Figures and Tables

**Table 1:** Predicted antigenic epitopes of VP2 protein (epitope marked with underline denotes the variant mutations).

Position	Sequence	Score
66–72	SRLVHLN	1.126
80–86	RRVVVNN	1.119
103–114	AQIVTPWSLVDA	1.128
126–131	WQLIVN	1.062
135–143	ELHLVSFEQ	1.135
145–156	IFNVVLKTVSES	1.179
170–178	TASLMVALD	1.121
195–202	LGFYPWKP	1.038
207–213	PWRYYFQ	1.037
219–227	MPSHTVPSN	1.078
247–256	ENSVPVHLLR	1.207
267–276	YFDCKPCRLT	1.134
285–296	LGLPPFLNSLPQ	1.120
335–346	EVGYSAPYYSFE	1.103
398–403	FTYIAR	1.038
421–434	NLPVTEDNVLLPTD	1.112
453–466	PLTALNNVPPVYPN	1.128
479–493	KPRLHVNAPFVCQNN	1.168
495–505	PGQLFVKVAPN	1.166
531–542	GKLVFKAKLRAS	1.128
558–565	QFNYVPSN	1.072
569–581	MKIVYEKSQLAPR	1.086

**Table 2:** Average *p*-value of recombination events from all detection methods.

Event	RDP	GENECONV	BootScan	MaxChi	Chimera	Siscan	3Seq
A	1.372 × 10^−12^	5.292 × 10^−12^	1.089 × 10^−15^	5.686 × 10^−13^	1.094 × 10^−11^	5.727 × 10^−17^	3.700 × 10^−20^
B	6.751 × 10^−9^	5.827 × 10^−9^	2.670 × 10^−9^	9.560 × 10^−10^	8.318 × 10^−8^	1.479 × 10^−16^	2.050 × 10^−1^
C	1.235 × 10^−13^	1.665 × 10^−11^	1.217 × 10^−8^	2.489 × 10^−10^	7.463 × 10^−10^	-	4.682 × 10^−21^
D	-	1.312 × 10^−7^	-	1.066 × 10^−12^	1.055 × 10^−12^	7.442 × 10^−18^	-
E	-	-	-	7.224 × 10^−6^	4.779 × 10^−4^	5.061 × 10^−13^	2.151 × 10^−9^
F	-	1.942 × 10^−4^	-	9.095 × 10^−4^	8.489 × 10^−4^	1.645 × 10^−5^	3.539 × 10^−1^
G	-	-	-	2.495 × 10^−1^	-	3.758 × 10^−6^	6.271 × 10^−9^
H	8.898 × 10^−2^	1.689 × 10^−3^	-	9.701 × 10^−4^	8.943 × 10^−4^	-	2.672 × 10^−6^
I	-	3.275 × 10^−3^	-	4.494 × 10^−3^	2.651 × 10^−3^	7.016 × 10^−6^	5.688 × 10^−7^

“-” indicates that the reorganization event was not detected using this detection method.

**Table 3:** The predicted results of each recombination event.

Recombination Event	Recombination Strain	Beginning Breakpoint	Ending Breakpoint	Major Parent		Minor Parent	
				Strain	Country	Strain	Country
A	AH2103	851	1424	HN2004	China	JS2007	China
B	JS2007	602	1181	AH2205	China	AH2203	China
C	HN2205	155	460	JS2001	China	JS2007	China
D	OQ868531	1182	437	HN2309	China	JS2007	China
E	JS2006	650	1676	HN2206	China	JS2104	China
F	AH2108	777	872	HB2002	China	AH2302	China
G	HN2206	668	1650	JS2205	China	AH2303	China
H	AH2108	426	651	AH2303	China	AH2103	China
I	AH2104	894	1181	EU914139	USA	AH2108	China

## Data Availability

All the data generated or analyzed during this study are included in this article. Datasets are deposited in a publicly accessible repository; the datasets generated for this study can be found in GenBank: https://www.ncbi.nlm.nih.gov/Genbank/ (accessed on 25 August 2024) (accessed on OR724741-OR724858). GenBank accession numbers are mentioned in the Materials and Methods of the article.

## Supplementary Materials

The following supporting information can be downloaded at: https://www.mdpi.com/article/10.3390/microorganisms12112173/s1, Supplementary Table S1. Information on Chinese Canine parvovirus type-2 (CPV-2) was sequenced in this study; Supplementary Table S2. Information on the canine parvovirus type-2 (CPV-2) reference sequences used in this study; Supplementary Table S3. Representative amino acid mutations in the CPV VP2 protein (the reference strain: Pfizer vaccine strain 06, accession number: FJ197846). Supplementary Material S2. The pairwise identities (%) matrix of VP2 of obtained strains and reference strains.
