# Supplementary material for: Genetic Diversity and Recombination Analysis of Canine Parvoviruses Prevalent in Central and Eastern China, from 2020 to 2023
Source: Microorganisms. 2024 Oct 29;12(11):2173. doi: 10.3390/microorganisms12112173 (PMC11596317; doi:10.3390/microorganisms12112173)
Supplement: Supplementary file 1 [file microorganisms-12-02173-s001.zip › Supplementary Tables.pdf]

Supplementary Material

Supplementary Table S1. Information on Chinese Canine parvovirus type-2 (CPV-2) was sequenced in this study.

Strain	Accession no.	Genotype	Province	Year	Host	Vaccination record	Age
JS2001	OR724741	CPV-2c	Jiangsu	2020	dog	2 doses	3 m
JS2002	OR724742	CPV-2c	Jiangsu	2020	dog	N.I.[a]	3 m
JS2003	OR724743	CPV-2c	Jiangsu	2020	dog	1 dose	1 m
JS2004	OR724744	CPV-2c	Jiangsu	2020	dog	2 doses	4 m
JS2005	OR724745	CPV-2c	Jiangsu	2020	dog	2 doses	3 m
JS2006	OR724746	new CPV-2a	Jiangsu	2020	dog	1 dose	2 m
JS2007	OR724747	CPV-2c	Jiangsu	2020	dog	N.I.	4 m
HB2001	OR724748	CPV-2	Hebei	2020	dog	2 doses	5 m
HB2002	OR724749	CPV-2c	Hebei	2020	dog	1 dose	1 m
HB2003	OR724750	CPV-2c	Hebei	2020	dog	1 dose	26 d
HB2004	OR724751	CPV-2c	Hebei	2020	dog	unvaccinated	1 m
HB2005	OR724752	new CPV-2a	Hebei	2020	dog	1 dose	2 m
HN2001	OR724753	CPV-2c	Henan	2020	dog	1 dose	2 m
HN2002	OR724754	CPV-2c	Henan	2020	dog	2 doses	4 m
HN2003	OR724755	CPV-2c	Henan	2020	dog	N.I.	3 m
HN2004	OR724756	CPV-2c	Henan	2020	dog	2 doses	3 m
HN2005	OR724757	CPV-2c	Henan	2020	dog	0 dose	2 m
HN2006	OR724758	CPV-2c	Henan	2020	dog	1 dose	1 m
HN2007	OR724759	CPV-2c	Henan	2020	dog	2 doses	4 m
HN2008	OR724760	CPV-2c	Henan	2020	dog	2 doses	5 m
AH2001	OR724761	CPV-2c	Anhui	2020	dog	2 doses	4 m
AH2002	OR724762	CPV-2c	Anhui	2020	dog	2 doses	3 m
AH2003	OR724763	new CPV-2a	Anhui	2020	dog	2 doses	6 m
AH2004	OR724764	new CPV-2b	Anhui	2020	dog	0 dose	1 m
AH2005	OR724765	CPV-2c	Anhui	2020	dog	2 doses	4 m
AH2006	OR724766	CPV-2c	Anhui	2020	dog	2 doses	7 m
AH2007	OR724767	CPV-2c	Anhui	2020	dog	2 doses	4 m
AH2008	OR724768	new CPV-2a	Anhui	2020	dog	1 dose	1 m
AH2009	OR724769	CPV-2c	Anhui	2020	dog	2 doses	5 m
AH2101	OR724770	CPV-2c	Anhui	2021	dog	1 dose	23 d
AH2102	OR724771	CPV-2c	Anhui	2021	dog	2 doses	4 m
AH2103	OR724772	CPV-2c	Anhui	2021	dog	2 doses	3 m
AH2104	OR724773	CPV-2c	Anhui	2021	dog	1 dose	2 m
AH2105	OR724774	CPV-2c	Anhui	2021	dog	2 doses	6 m
AH2106	OR724775	CPV-2c	Anhui	2021	dog	2 doses	4 m

AH2107	OR724776	new CPV-2a	Anhui	2021	dog	2 doses	3 m
AH2108	OR724777	new CPV-2a	Anhui	2021	dog	N.I.	2 m
AH2109	OR724778	new CPV-2a	Anhui	2021	dog	2 doses	2 m
AH2110	OR724779	CPV-2c	Anhui	2021	dog	2 doses	4 m
HN2101	OR724780	CPV-2c	Henan	2021	dog	2 doses	5 m
HN2102	OR724781	CPV-2c	Henan	2021	dog	N.I.	4 m
HN2103	OR724782	CPV-2c	Henan	2021	dog	1 dose	1 m
HN2104	OR724783	CPV-2c	Henan	2021	dog	1 dose	2 m
HN2105	OR724784	CPV-2c	Henan	2021	dog	2 doses	3 m
HN2106	OR724785	CPV-2c	Henan	2021	dog	2 doses	5 m
JS2101	OR724786	CPV-2c	Jiangsu	2021	dog	1 dose	20 d
JS2102	OR724787	CPV-2c	Jiangsu	2021	dog	2 doses	3 m
JS2103	OR724788	CPV-2c	Jiangsu	2021	dog	2 doses	3 m
JS2104	OR724789	CPV-2c	Jiangsu	2021	dog	2 doses	4 m
JS2105	OR724790	CPV-2c	Jiangsu	2021	dog	2 doses	3 m
JS2106	OR724791	CPV-2c	Jiangsu	2021	dog	1 dose	1 m
HB2101	OR724792	CPV-2c	Hebei	2021	dog	1 dose	4 d
HB2102	OR724793	CPV-2c	Hebei	2021	dog	1 dose	26 d
HB2103	OR724794	CPV-2c	Hebei	2021	dog	unvaccinated	2 m
HB2104	OR724795	CPV-2c	Hebei	2021	dog	2 doses	3 m
HB2105	OR724796	CPV-2c	Hebei	2021	dog	2 doses	4 m
HB2106	OR724797	CPV-2c	Hebei	2021	dog	2 doses	6 m
HB2107	OR724798	CPV-2c	Hebei	2021	dog	2 doses	4 m
HB2108	OR724799	CPV-2c	Hebei	2021	dog	1 dose	1 m
AH2201	OR724800	CPV-2c	Anhui	2022	dog	N.I.	2 m
AH2202	OR724801	CPV-2c	Anhui	2022	dog	2 doses	3 m
AH2203	OR724802	new CPV-2a	Anhui	2022	dog	unvaccinated	21 d
AH2204	OR724803	CPV-2c	Anhui	2022	dog	1 dose	1 m
AH2205	OR724804	CPV-2c	Anhui	2022	dog	2 doses	2 m
AH2206	OR724805	CPV-2c	Anhui	2022	dog	2 doses	4 m
AH2207	OR724806	CPV-2c	Anhui	2022	dog	2 doses	4 m
AH2208	OR724807	new CPV-2a	Anhui	2022	dog	1 dose	2 m
HB2201	OR724808	CPV-2c	Hebei	2022	dog	1 dose	1 m
HB2202	OR724809	CPV-2c	Hebei	2022	dog	unvaccinated	3 m
HB2203	OR724810	CPV-2c	Hebei	2022	dog	unvaccinated	1 m
HB2204	OR724811	CPV-2c	Hebei	2022	dog	2 doses	3 m
HB2205	OR724812	CPV-2c	Hebei	2022	dog	2 doses	5 m
HB2206	OR724813	CPV-2c	Hebei	2022	dog	N.I.	5 m
HB2207	OR724814	CPV-2c	Hebei	2022	dog	1 dose	1 m
JS2201	OR724815	CPV-2c	Jiangsu	2022	dog	2 doses	3 m
JS2202	OR724816	CPV-2c	Jiangsu	2022	dog	2 doses	3 m
JS2203	OR724817	CPV-2c	Jiangsu	2022	dog	2 doses	2 m
JS2204	OR724818	CPV-2c	Jiangsu	2022	dog	1 dose	18 d
JS2205	OR724819	new CPV-2a	Jiangsu	2022	dog	1 dose	1 m

JS2206	OR724820	CPV-2c	Jiangsu	2022	dog	2 doses	4 m
JS2207	OR724821	CPV-2c	Jiangsu	2022	dog	2 doses	2 m
JS2208	OR724822	CPV-2c	Jiangsu	2022	dog	2 doses	3 m
HN2201	OR724823	CPV-2c	Henan	2022	dog	2 doses	2 m
HN2202	OR724824	CPV-2c	Henan	2022	dog	N.I.	4 m
HN2203	OR724825	CPV-2c	Henan	2022	dog	1 dose	2 m
HN2204	OR724826	CPV-2c	Henan	2022	dog	1 dose	1 m
HN2205	OR724827	CPV-2c	Henan	2022	dog	2 doses	3 m
HN2206	OR724828	CPV-2c	Henan	2022	dog	2 doses	2 m
HN2207	OR724829	CPV-2c	Henan	2022	dog	2 doses	4 m
HN2208	OR724830	CPV-2c	Henan	2022	dog	1 dose	1 m
HN2209	OR724831	CPV-2c	Henan	2022	dog	2 doses	3 m
HN2210	OR724832	CPV-2c	Henan	2022	dog	1 dose	2 m
HN2301	OR724833	CPV-2c	Henan	2023	dog	unvaccinated	2 m
HN2302	OR724834	CPV-2c	Henan	2023	dog	1 dose	3 m
HN2303	OR724835	CPV-2c	Henan	2023	dog	2 doses	3 m
HN2304	OR724836	CPV-2c	Henan	2023	dog	2 doses	2 m
HN2305	OR724837	CPV-2c	Henan	2023	dog	2 doses	2 m
HN2306	OR724838	CPV-2c	Henan	2023	dog	1 dose	1 m
HN2307	OR724839	CPV-2c	Henan	2023	stray dog	N.I.	N.I.
HN2308	OR724840	CPV-2c	Henan	2023	stray dog	N.I.	N.I.
HN2309	OR724841	CPV-2c	Henan	2023	stray dog	N.I.	N.I.
HN2310	OR724842	CPV-2c	Henan	2023	stray dog	N.I.	N.I.
HN2311	OR724843	new CPV-2b	Henan	2023	stray dog	N.I.	N.I.
JS2301	OR724844	CPV-2c	Jiangsu	2023	dog	1 dose	1 m
JS2302	OR724845	CPV-2c	Jiangsu	2023	dog	2 doses	4 m
JS2303	OR724846	CPV-2c	Jiangsu	2023	dog	1 dose	2 m
JS2304	OR724847	CPV-2c	Jiangsu	2023	dog	2 doses	3 m
JS2305	OR724848	CPV-2c	Jiangsu	2023	dog	1 dose	1 m
JS2306	OR724849	CPV-2c	Jiangsu	2023	dog	1 dose	1 m
AH2301	OR724850	CPV-2c	Anhui	2023	dog	2 doses	3 m
AH2302	OR724851	CPV-2c	Anhui	2023	dog	2 doses	2 m
AH2303	OR724852	CPV-2c	Anhui	2023	dog	2 doses	5 m
AH2304	OR724853	CPV-2c	Anhui	2023	dog	N.I.	3 m
AH2305	OR724854	CPV-2c	Anhui	2023	dog	1 dose	2 m
HB2301	OR724855	CPV-2c	Hebei	2023	dog	1 dose	2 m
HB2302	OR724856	CPV-2c	Hebei	2023	dog	2 doses	3 m
HB2303	OR724857	CPV-2c	Hebei	2023	dog	unvaccinated	4 m
HB2304	OR724858	CPV-2c	Hebei	2023	dog	2 doses	3 m

CPV-2: Canine parvovirus type-2,[a] no information.

Supplementary Table S2. Information on the canine parvovirus type-2 (CPV-2) reference sequences used in this study.

Strain	Accession No.	Genotype	Regions	Year
CPV-b	M38245	CPV-2	The USA	1990
CPVint (vaccine)	FJ197846	CPV-2	South Korea	2007
Pfizer/vaccine/06	EU914139	CPV-2	The USA	2008
CPV-15	M24003	CPV-2a	The USA	1993
CPV-39	M74849	CPV-2b	The USA	1993
CPV-W42	AF306444	CPV-2b	Italy	2001
CPV-339	AY742933	new CPV-2a	New Zealand	1993
Henan42	KJ438805	new CPV-2a	China	2013
GY-3	KY386852	new CPV-2a	China	2015
02B9	DQ025950	new CPV-2a	France	2005
K022	EU009203	new CPV-2a	South Korea	2007
LZ2	JQ268284	new CPV-2b	China	2011
BM-11	JQ743894	new CPV-2b	China	2011
Wuhan2	KC881278	new CPV-2b	China	2010
LCPV V204	AB054221	new CPV-2b	China	2001
DH326	EF599097	new CPV-2b	South Korea	2007
HRB-A6	KT156832	CPV-2c	China	2014
06-09	GU380303	CPV-2c	China	2009
G367-97	FJ005202	CPV-2c	Germany	1997
ME10	KF149963	CPV-2c	Ecuador	2012
ME28	KF149984	CPV-2c	Ecuador	2012
G333-99	FJ005204	CPV-2c	Germany	1999
M124	KC196108	CPV-2c	Uruguay	2008
ME1	KF149962	CPV-2c	Ecuador	2012
2c-MUT14	ON323043	CPV-2c	Thailand	2019
2c-MUT11	ON323042	CPV-2c	Thailand	2019
HN1911	OQ868522	CPV-2c	China	2019
JS1901	OQ868523	CPV-2c	China	2019
JS1902	OQ868524	CPV-2c	China	2019
HN1907	OQ868525	CPV-2c	China	2019
AH2005	OQ868526	new CPV-2a	China	2020
AH2008	OQ868527	CPV-2c	China	2020
JS2101	OQ868528	CPV-2c	China	2021
HN2102	OQ868529	new CPV-2a	China	2021
HN2104	OQ868530	CPV-2c	China	2021
HN2103	OQ868531	CPV-2c	China	2021
HN2201	OQ868532	CPV-2c	China	2022

Supplementary Table S3. Representative amino acid mutations in the CPV VP2 protein (the reference strain: Pfizer vaccine strain 06, accession number: FJ197846).

Strain	Mutation sites														
	5	87	101	219	267	297	300	305	324	370	375	386	426	440	447

FJ197846	A	M	I	V	F	S	A	D	Y	Q	N	K	N	T	I
JS2001	G	L	T	M	Y	A	G	Y	I	R	D	Q	E	-	-
JS2002	G	L	T	I	Y	A	G	Y	I	R	D	Q	E	-	-
JS2003	G	L	T	I	Y	A	G	Y	I	R	D	Q	E	-	-
JS2004	G	L	T	I	Y	A	G	Y	I	R	D	Q	E	-	-
JS2005	G	L	T	I	Y	A	G	Y	I	R	D	Q	E	-	-
JS2006	-	L	T	I	Y	A	G	Y	I	-	D	Q	-	A	-
JS2007	G	L	T	I	Y	A	G	Y	I	R	D	Q	E	-	-
HB2001	G	L	T	I	Y	A	G	Y	I	R	D	Q	E	-	-
HB2002	G	L	T	I	Y	A	G	Y	I	R	D	Q	E	-	-
HB2003	G	L	T	I	Y	A	G	Y	I	R	D	Q	E	-	-
HB2004	G	L	T	I	Y	A	G	Y	I	R	D	Q	E	-	-
HB2005	-	L	T	I	Y	A	G	Y	I	-	D	Q	-	A	-
HN2001	G	L	T	I	Y	A	G	Y	I	R	D	Q	E	-	-
HN2002	G	L	T	I	Y	A	G	Y	I	R	D	Q	E	-	-
HN2003	G	L	T	I	Y	A	G	Y	I	R	D	Q	E	-	-
HN2004	G	L	T	I	Y	A	G	Y	I	R	D	Q	E	-	-
HN2005	G	L	T	I	Y	A	G	Y	I	R	D	Q	E	-	-
HN2006	G	L	T	I	Y	A	G	Y	I	R	D	Q	E	-	-
HN2007	G	L	T	I	Y	A	G	Y	I	R	D	Q	E	-	-
HN2008	G	L	T	I	Y	A	G	Y	I	R	D	Q	E	-	-
AH2001	G	L	T	I	Y	A	G	Y	I	R	D	Q	E	-	-
AH2002	G	L	T	I	Y	A	G	Y	I	R	D	Q	E	-	-
AH2003	-	L	T	I	Y	A	G	Y	I	-	D	Q	-	A	-
AH2004	-	L	T	I	Y	A	G	Y	I	-	D	Q	D	A	-
AH2005	G	L	T	I	Y	A	G	Y	I	R	D	Q	E	-	-
AH2006	G	L	T	I	Y	A	G	Y	I	R	D	Q	E	-	-
AH2007	G	L	T	I	Y	A	G	Y	I	R	D	Q	E	-	-
AH2008	-	L	T	I	Y	A	G	Y	I	-	D	Q	-	A	-
AH2009	G	L	T	I	Y	A	G	Y	I	R	D	Q	E	-	-
AH2101	G	L	T	I	Y	A	G	Y	I	R	D	Q	E	-	-
AH2102	G	L	T	I	Y	A	G	Y	I	R	D	Q	E	-	-
AH2103	G	L	T	I	Y	A	G	Y	H	R	D	Q	E	-	-
AH2104	G	L	T	I	Y	A	G	Y	H	R	D	Q	E	-	-
AH2105	G	L	T	I	Y	A	G	Y	I	R	D	Q	E	-	-
AH2106	G	L	T	I	Y	A	V	I	I	R	D	Q	E	-	-
AH2107	-	L	T	I	L	A	G	Y	I	-	D	Q	-	A	-
AH2108	-	L	T	I	-	A	-	-	I	-	D	Q	-	-	-
AH2109	-	L	T	I	Y	A	G	Y	I	-	D	Q	-	A	-
AH2110	G	L	T	I	Y	A	G	Y	I	R	D	Q	E	-	-
HN2101	G	L	T	I	Y	A	G	Y	I	R	D	Q	E	-	-
HN2102	G	L	T	I	Y	A	G	Y	I	R	D	Q	E	-	-
HN2103	G	L	T	I	Y	A	G	Y	I	R	D	Q	E	-	-
HN2104	G	L	T	I	Y	A	G	Y	I	R	D	Q	E	-	-

HN2105	G	L	T	I	Y	A	G	Y	I	R	D	Q	E	-	-
HN2106	G	L	T	I	Y	A	G	Y	I	R	D	Q	E	-	-
JS2101	G	L	T	I	Y	A	G	Y	I	R	D	Q	E	-	-
JS2102	G	L	T	I	Y	A	G	Y	I	R	D	Q	E	-	-
JS2103	G	L	T	I	Y	A	G	Y	I	R	D	Q	E	-	-
JS2104	G	L	T	I	Y	A	G	Y	I	-	D	Q	E	-	-
JS2105	G	L	T	I	Y	A	G	Y	I	R	D	Q	E	-	-
JS2106	G	L	T	I	Y	A	G	Y	I	R	D	Q	E	-	-
HB2101	G	L	T	I	Y	A	G	Y	I	R	D	Q	E	-	-
HB2102	G	L	T	I	Y	A	G	Y	I	R	D	Q	E	-	-
HB2103	G	L	T	I	Y	A	G	Y	I	R	D	Q	E	-	-
HB2104	G	L	T	I	Y	A	G	Y	I	R	D	Q	E	-	-
HB2105	-	L	T	I	Y	A	G	Y	I	R	D	Q	E	-	-
HB2106	G	L	T	I	Y	A	G	Y	I	R	D	Q	E	-	-
HB2107	G	L	T	I	Y	A	G	Y	I	R	D	Q	E	-	-
HB2108	G	L	T	I	-	A	G	Y	I	R	D	Q	E	-	-
AH2201	G	L	T	I	Y	A	G	Y	I	R	D	Q	E	-	-
AH2202	G	L	T	I	Y	A	G	Y	I	R	D	Q	E	-	-
AH2203	-	L	T	I	Y	A	G	Y	I	-	D	Q	-	A	-
AH2204	G	L	T	I	Y	A	G	Y	I	R	D	Q	E	-	-
AH2205	-	L	T	I	Y	A	G	Y	I	R	D	Q	E	-	-
AH2206	G	L	T	I	Y	A	G	Y	I	R	D	Q	E	-	-
AH2207	-	L	T	I	Y	A	G	Y	I	R	D	Q	E	-	-
AH2208	-	L	T	I	Y	A	G	Y	I	-	D	Q	-	A	-
HB2201	G	L	T	I	Y	A	G	Y	I	R	D	Q	E	-	-
HB2202	G	L	T	I	Y	A	G	Y	I	R	D	Q	E	-	-
HB2203	G	L	T	I	Y	A	G	Y	I	R	D	Q	E	-	-
HB2204	G	L	T	I	Y	A	G	Y	I	R	D	Q	E	-	-
HB2205	G	L	T	I	Y	A	G	Y	I	R	D	Q	E	-	-
HB2206	G	L	T	I	Y	A	G	Y	I	R	D	Q	E	-	-
HB2207	-	L	T	I	Y	A	G	Y	I	R	D	Q	E	-	-
JS2201	G	L	T	I	Y	A	G	Y	I	R	D	Q	E	-	-
JS2202	G	L	T	I	Y	A	G	Y	I	R	D	Q	E	-	-
JS2203	G	L	T	I	Y	A	G	Y	I	R	D	Q	E	-	-
JS2204	G	L	T	I	Y	A	G	Y	I	R	D	Q	E	-	-
JS2205	G	L	T	I	Y	A	G	Y	I	R	D	Q	-	-	-
JS2206	-	L	T	I	Y	A	G	Y	I	R	D	Q	E	-	-
JS2207	G	L	T	I	Y	A	G	Y	I	R	D	Q	E	-	-
JS2208	G	L	T	I	Y	A	G	Y	I	R	D	Q	E	-	-
HN2201	G	L	T	I	Y	A	G	Y	I	R	D	Q	E	-	-
HN2202	-	L	T	I	Y	A	G	Y	I	R	D	Q	E	-	-
HN2203	G	L	T	I	Y	A	G	Y	I	R	D	Q	E	-	-
HN2204	G	L	T	I	Y	A	G	Y	I	R	D	Q	E	-	-
HN2205	G	L	T	I	Y	A	G	Y	I	R	D	Q	E	-	-

HN2206	G	L	T	I	Y	A	G	Y	I	R	D	Q	E	-	-
HN2207	G	L	T	I	Y	A	G	Y	I	R	D	Q	E	-	-
HN2208	G	L	T	I	Y	A	G	Y	I	R	D	Q	E	-	-
HN2209	G	L	T	I	Y	A	G	Y	I	R	D	Q	E	-	-
HN2210	G	L	T	I	Y	A	G	Y	I	R	D	Q	E	-	-
HN2301	-	L	T	I	Y	A	G	Y	I	R	D	Q	E	-	-
HN2302	-	L	T	I	Y	A	G	Y	I	R	D	Q	E	-	-
HN2303	-	L	T	I	Y	A	G	Y	I	R	D	Q	E	-	M
HN2304	G	L	T	I	Y	A	G	Y	I	R	D	Q	E	-	-
HN2305	G	L	T	I	Y	A	G	Y	I	R	D	Q	E	-	-
HN2306	-	L	T	I	Y	A	G	Y	I	R	D	Q	E	-	-
HN2307	G	L	T	I	Y	A	G	Y	I	R	D	Q	E	-	-
HN2308	G	L	T	I	Y	A	G	Y	I	R	D	Q	E	-	-
HN2309	G	L	T	I	Y	A	G	Y	I	R	D	Q	E	-	-
HN2310	-	L	T	I	Y	A	G	Y	I	R	D	Q	E	-	-
HN2311	-	L	T	I	Y	A	G	Y	I	R	D	Q	D	A	-
JS2301	G	L	T	I	Y	A	G	Y	I	R	D	Q	E	-	M
JS2302	G	L	T	I	Y	A	G	Y	I	R	D	Q	E	-	-
JS2303	G	L	T	I	Y	A	G	Y	I	R	D	Q	E	-	-
JS2304	G	L	T	I	Y	A	G	Y	I	R	D	Q	E	-	-
JS2305	-	L	T	I	Y	A	G	Y	I	R	D	Q	E	-	-
JS2306	G	L	T	I	Y	A	G	Y	I	R	D	Q	E	-	-
AH2301	G	L	T	I	Y	A	G	Y	I	R	D	Q	E	-	M
AH2302	G	L	T	I	-	A	G	Y	I	R	D	Q	E	-	-
AH2303	-	L	T	I	Y	A	G	Y	I	R	D	Q	E	-	-
AH2304	G	L	T	I	Y	A	G	Y	I	R	D	Q	E	-	-
AH2305	G	L	T	I	Y	A	G	Y	I	R	D	Q	E	-	-
HB2301	G	L	T	I	Y	A	G	Y	I	R	D	Q	E	-	M
HB2302	G	L	T	I	Y	A	G	Y	I	R	D	Q	E	-	-
HB2303	G	L	T	I	Y	A	G	Y	I	R	D	Q	E	-	-
HB2304	G	L	T	I	Y	A	G	Y	I	R	D	Q	E	-	M

"-" indicates that the location of the sequenced strain was the same as the reference strain, and the underlined strains were obtained from the Chinese CPV-2 strain. (A: Ala, D: Asp, E: Glu, F: Phe, I: Ile, G: Gly, K: Lys, L: Leu, M: Met, N: Asn, P: Pro, Q: Gln, R: Arg, S: Ser, T: Thr, V: Val, Y: Tyr).
